# Distributed Cortical Regions for the Recall of People, Places, and Objects

**DOI:** 10.1523/ENEURO.0496-24.2024

**Published:** 2025-01-10

**Authors:** Alexis Kidder, Edward H. Silson, Matthias Nau, Chris I. Baker

**Affiliations:** ^1^Department of Psychological and Brain Sciences, Dartmouth College, Hanover, New Hampshire 03755; ^2^Section on Learning and Plasticity, Laboratory of Brain and Cognition, National Institute of Mental Health, Bethesda, Maryland 20892-1366; ^3^Department of Psychology, School of Philosophy, Psychology and Language Sciences, The University of Edinburgh, Edinburgh EH8 9JZ, United Kingdom

**Keywords:** fMRI, memory recall, objects, people, places

## Abstract

The human medial parietal cortex (MPC) is recruited during multiple cognitive processes. Previously, we demonstrated regions specific to recall of people or places and proposed that the functional organization of MPC mirrors the category selectivity defining the medial–lateral axis of the ventral–temporal cortex (VTC). However, prior work considered recall of people and places only, and VTC also shows object selectivity sandwiched between face- and scene-selective regions. Here, we tested a strong prediction of our proposal: like VTC, MPC should show a region specifically recruited during object recall, and its relative cortical position should mirror the one of VTC. While responses during people and place recall showed a striking replication of prior findings, we did not observe any evidence for object-recall effects within MPC, which differentiates it from the spatial organization in VTC. Importantly, beyond MPC, robust recall effects were observed for people, places, and objects on the lateral surface of the brain. Place-recall effects were present in the angular gyrus, frontal eye fields, and peripheral portions of the early visual cortex, whereas people recall selectively drove response in the right posterior superior temporal sulcus. Object-recall effects were largely restricted to a region posterior to the left somatosensory cortex, in the vicinity of the supramarginal gyrus. Taken together, these data demonstrate that while there are distributed regions active during recall of people, places, and objects, the functional organization of MPC does not mirror the medial–lateral axis of VTC but reflects only the most salient features of that axis—namely, representations of people and places.

## Significance Statement

The human medial parietal cortex (MPC) is recruited during multiple cognitive processes. Recently, we proposed a framework for interpreting the functional organization of MPC by suggesting that it reflects the categorical preferences for people and places that is evident also in the ventral–temporal cortex (VTC). Because VTC also exhibits selectivity for objects, we here extend this framework to test whether MPC also shows object selectivity during recall. Robust people and place-recall effects were evident in MPC, but we found no evidence for object recall within MPC, suggesting that MPC and VTC are not mirror-copies of each other. Together, these data suggest that the functional organization of MPC reflects the most salient categorical representations within VTC for people and places.

## Introduction

The human medial parietal cortex (MPC) is associated with a broad array of cognitive functions, including memory recall (Wagner et al., 2005; Vilberg and Rugg, 2008; Kim, 2013; Gilmore et al., 2015; Silson et al., 2019a,b; Steel et al., 2021), visual scene perception (Baldassano et al., 2013; Epstein and Baker, 2019; Silson et al., 2019a), scene construction (Hassabis et al., 2007), processing of spatial and other contextual associations (Bar and Aminoff, 2003), navigation (Epstein, 2008; Epstein and Baker, 2019), future thinking (Szpunar et al., 2007; Benoit and Schacter, 2015; Gilmore et al., 2018), and mental orientation (Peer et al., 2015).

**Figure 1.**
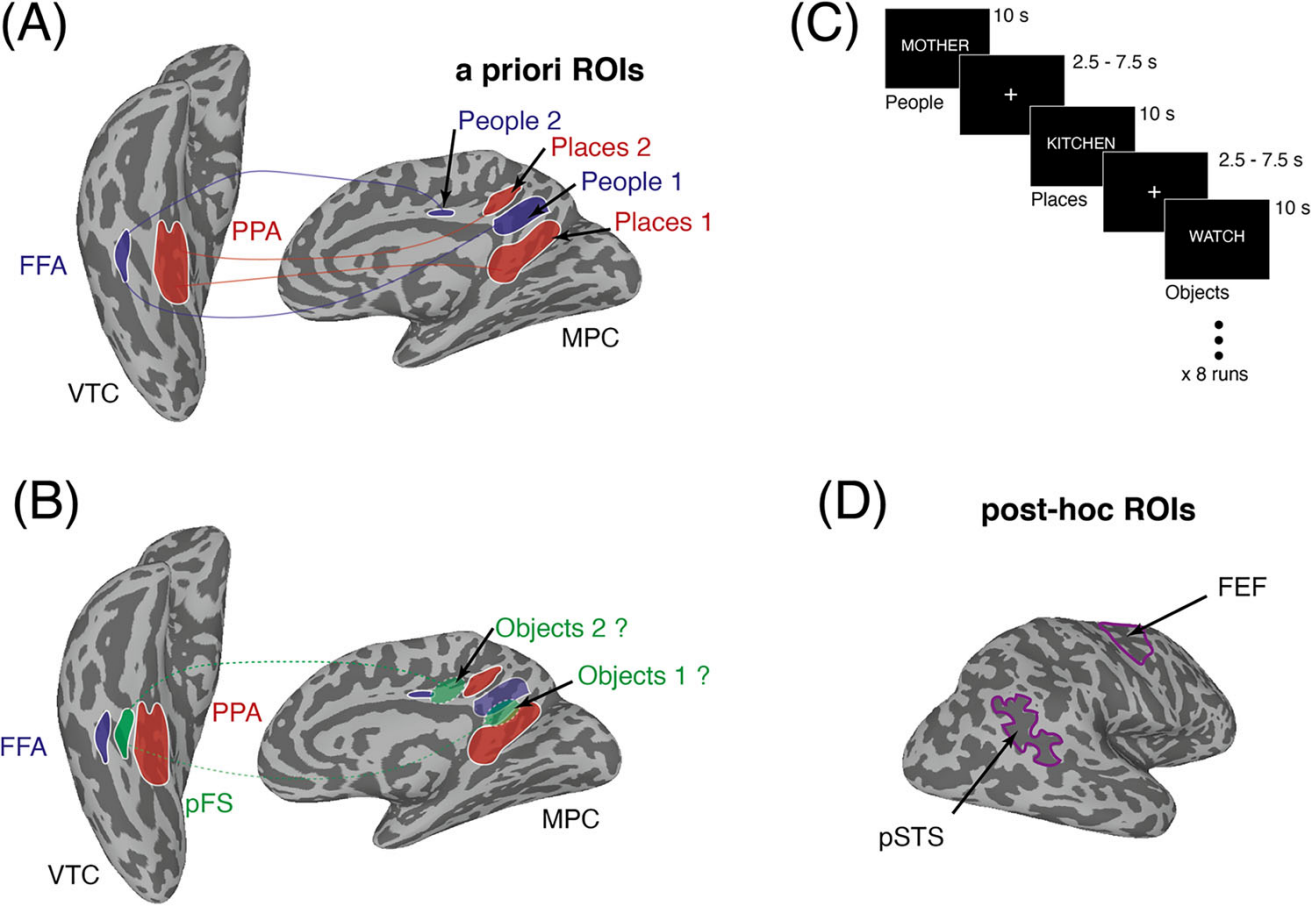
Functional link between VTC and MPC, predictions, task schematic, and post hoc ROIs. ***A***, Partially inflated ventral and medial views of the right hemisphere of a representative participant are shown (light gray, gyri; dark gray, sulci). Overlaid in false color onto the ventral surface are group-based ROIs for face-selective FFA (blue) and scene-selective PPA (red). Overlaid in false color onto the medial surface are group-based ROIs for people and place recall areas within MPC (People 1/People 2, blue; Places 1/Places 2, red) taken from our prior work (Silson et al., 2019b). Along the posterior/ventral–anterior/dorsal axis of MPC, these regions show an alternating pattern (Places, People, Places, People). Lines connecting VTC with MPC illustrate the consistent category preferences. ***B***, The same ventral and medial views of the right hemisphere in ***A*** are shown. Overlaid in false color onto the ventral surface is the group-based ROI for object-selective pFS (green). Within VTC, object-selective responses (pFS) are sandwiched between face-selective responses (FFA) more laterally and scene-selective responses (PPA) more medially. If the functional organization of MPC reflects that of VTC, we predict object-recall areas to be located in-between areas recruited during people and place recall. This predicted pattern of results in overload in false color onto the medial surface. Green-dashed lines depict the hypothesized link with pFS on the ventral surface. ***C***, Task schematic. During each trial, participants were instructed to recall from memory either personally familiar people (e.g., mother), places (e.g., kitchen), or objects (e.g., watch). Participants were instructed to visualize the target as vividly as possible for the duration of the trials (10 s). Trials were separated by a variable intertrial interval (2.5–7.5 s). Each run contained six randomized trials from each category. Participants completed eight runs of the experiment. ***D***, Overlaid onto a lateral view of the right hemisphere are the locations of two post hoc ROIs (purple outlines), the pSTS was taken from an independent group-based localizer (see Materials and Methods). FEF were defined according to a probabilistic retinotopic atlas (Wang et al., 2015).

The diversity with which MPC is recruited has prompted recent work from our group (Silson et al., 2019b; Bainbridge et al., 2021; Steel et al., 2021) and others (Peer et al., 2015; Chrastil, 2018; Woolnough et al., 2020; Deen and Freiwald, 2021) to explore whether there is an underlying functional organization to MPC. Specifically, in a series of experiments, we identified a functional link between the medial–lateral axis of the ventral–temporal cortex (VTC) and the posterior/ventral–anterior/dorsal axis of MPC (Silson et al., 2019b). First, distinct regions of MPC were identified based on patterns of differential connectivity with face- and scene-selective regions, respectively. Second, we found that these same regions responded most strongly when processing visual stimuli of their preferred category. Finally, via cued recall of either people or places, we demonstrated an alternating pattern of recruitment along the posterior/ventral–anterior/dorsal axis of MPC. These data led to the suggestion that the functional organization of MPC may be a recapitulation of the medial–lateral axis of VTC (Fig. 1*A*), in which visual face selectivity (i.e., fusiform face area, FFA) and visual scene selectivity (i.e., parahippocampal place area, PPA; Fig. 1*B*; Kanwisher and Dilks, 2013; Weiner et al., 2014; Epstein and Baker, 2019) are located in distinct, yet adjacent regions. Importantly, VTC also contains a region that has been characterized as object-selective (i.e., posterior fusiform cortex, pFS), in-between FFA and PPA. Here, we tested a strong prediction of our framework and investigated whether there is also an object-selective region in MPC during memory recall.

Participants recalled personally familiar people, places, or objects, allowing us to accomplish two goals. First, it provided an opportunity for a direct, yet independent test of prior work on recall of people and places in MPC (Silson et al., 2019b). Second, it allowed us to examine whether or not the recall of personally familiar objects recruited distinct regions of MPC, akin to the perception of objects in VTC (i.e., pFS). Given the stereotypical location of pFS relative to both FFA and PPA in VTC (Kanwisher and Dilks, 2013), we hypothesized that if the recall of objects recruited distinct regions of MPC, these would likely fall between those recruited during place and people recall (Silson et al., 2019b; Fig. 1*B*).

Recall of people and places produced a pattern of recruitment within MPC that was largely indistinguishable from prior work (Silson et al., 2019b) and constitutes a complete yet independent replication. However, we found no evidence for distinct responses during object recall within MPC, despite strong predictions based on the functional organization of VTC. However, we did observe object-recall effects outside MPC, in a region posterior and inferior of the primary somatosensory cortex in the vicinity of the supramarginal gyrus. Moreover, people recall selectively drove responses in the right-hemispheric posterior superior temporal sulcus (pSTS), whereas place recall recruited the bilateral angular gyrus, peripheral portions of the early visual cortex (EVC), and frontal eye fields (FEF). Taken together, these data highlight distributed regions preferentially engaged during recall and suggest that, in contrast to VTC, the functional organization of MPC is largely dominated by representations of people and places, whereas object recall engages action-related areas.

## Materials and Methods

### Participants

Twenty-four participants (17 females; mean age, 24.2 years; 21–35) were recruited from the DC area and National Institute of Health (NIH) community. All participants were right-handed with normal or corrected-to-normal vision and neurologically healthy. All participants gave written informed consent according to procedures approved by the NIH Institutional Review Board (protocol 93M-0170, clinical trials #NCT00001360). Participants were compensated monetarily for their time. The sample size for the memory experiment was based on prior work from our group employing a very similar paradigm (Silson et al., 2019a,b), where sample sizes were *n* = 19 and *n* = 24, respectively. Due to excessive motion during scanning, only 20 out of the 24 datasets were analyzed in full.

### Stimuli and tasks

#### Memory experiment

Experimental details were based on prior work (Silson et al., 2019b) and stimuli consisted of written names of 12 personally familiar people (e.g., mother), 12 personally familiar places (e.g., kitchen), and 12 personally familiar objects (e.g., watch). Importantly, these objects were specific to the participant. Participants were instructed to recall their specific target object (e.g., their watch) and not a generic exemplar (e.g., generic watch). The stimuli were provided by participants through a survey completed prior to the fMRI scan. Importantly, we selected objects that did not have strong contextual associations to certain places/scenes (e.g., wallet), as contextual association is thought to drive responses in certain MPC regions (Bar and Aminoff, 2003). Word stimuli were presented in white 18-point Arial font, all capital type against a black background. During each trial of the task (Fig. 1*C*), participants were instructed to visualize the named item from memory as vividly as possible for the duration of the trial (10 s). Mean character length: objects, 6.31; places, 12.05; and people, 6.42. Trials were separated by a variable intertrial interval (2.5–7.5 s). In each of the eight runs, there were six trials of each condition (people, places, objects) presented in a randomized order, for a total of 18 trials per run (144 trials total). Across the entire scanning session, each stimulus was presented four times.

#### Postscan questionnaire

After the scan, participants completed a questionnaire in which they rated how vividly they were able to visualize from memory each item named during the memory runs. The stimuli were listed in the same order they appeared during the memory experiment and were rated on a four-point Likert–type scale (1, not at all vivid; 4, extremely vivid). If the participant could not visualize the stimulus at all while in the scanner, they checked a box on the questionnaire.

### Functional imaging parameters

#### Memory experiment

All scans were performed on a 3.0 T GE 750 MRI scanner using a 32-channel head coil. All functional images were acquired using a BOLD contrast-sensitive three–echo echoplanar sequence (ASSET acceleration factor, 2; TEs, 12.5, 27.7, and 42.9 ms; flip angle, 75°; 64 × 64 matrix; in-plane resolution, 3.2 × 3.2 mm; slice thickness, 3.5 mm; TR, 2,500 ms; 35 slices).

#### fMRI data preprocessing

Data were preprocessed using AFNI (Cox, 1996; RRID: SCR_005927). The first four volumes of each run were discarded to allow for T1 equilibration effects. Initial preprocessing steps for fMRI data were carried out on each echo separately. Slice-time correction was applied (3dTShift), and signal outliers were attenuated (3dDespike). Motion-correction parameters were estimated from the middle echo based on rigid-body registration of each volume to the first volume of the scan; these alignment parameters were then applied to all echos. Data from all three acquired echoes were then registered to each participants’ T1 image and combined to remove additional thermal and physiological noise using multiecho independent components analysis (ME-ICA; Kundu et al., 2012, 2013). This procedure computes a weighted-average of the three echo times for each scan run to reduce thermal noise within each voxel. It subsequently performs a spatial ICA to identify and remove noise components from the data. This is accomplished by comparing each component to a model that assumes a temporal dependence in signal decay (i.e., “BOLDlike”) and to a different model that assumes temporal independence (i.e., “non-BOLD-like”). Components with a strong fit to the former and a poor fit to the latter are retained for subsequent analysis. This procedure was conducted using default options in AFNI's tedana.py function. ME-ICA-processed data from each scan were then aligned across runs for each participant.

#### Memory analysis

Analyses were conducted using a general linear model and the AFNI programs 3dDeconvolve and 3dREMLfit. The data at each time point were treated as the sum of all effects thought to be present, and the time series was compared with a generalized least squared regression model fit with REML estimation of the temporal autocorrelation structure. Responses were modeled by convolving a standard gamma function with a 10 s square wave for each condition of interest (people, places, objects). Estimated motion parameters were included as additional regressors of noninterest, and fourth-order polynomials were included to account for slow drifts in the MR signal over time. Significance was determined by comparing the beta estimates for each condition (normalized by the grand mean of each voxel for each run) against the baseline (fixation only).

#### Sampling of data to the cortical surface

In each participant, statistical datasets were projected onto surface reconstructions of each individual participant's hemispheres using the Surface Mapping with AFNI (SUMA) software (Saad and Reynolds, 2012). First, data were aligned to high-resolution anatomical scans (align_epi_anat.py). Once aligned, these data were projected onto the cortical surface (3dVol2Surf) and smoothed with a 2 mm full-width at half-maximum 2D Gaussian kernel.

#### Cortical regions of interest

Initially, we utilized the whole-brain data from prior work (Silson et al., 2019b) to define four regions of interest (ROIs) within MPC of each hemisphere (Places1, People1, Places2, and People2; Fig. 1*A*). Importantly, these data were acquired in an independent group of participants. The FEF were defined using a probabilistic retinotopic atlas (Wang et al., 2015). In addition, we made use of a group-based (*n* = 15) dynamic localizer using the contrast of faces > objects from an unpublished dataset in independent participants to localize face-selective pSTS.

#### Eyeball-voxel analysis

To test whether recall-related eye movements may have contributed to our imaging results, we performed multivariate pattern analysis (MVPA) using the multivoxel pattern of the eyeballs. To do so, we identified the eyeball voxels in our images following established approaches (Frey et al., 2021), which entailed coregistering the eyeballs to the ones of a template in which the eyes were manually delineated. This dedicated eyeball coregistration procedure ensured that the eyeball voxels were correctly identified and that the corresponding multivoxel pattern could be extracted from each functional volume [see Frey et al. (2021) for more details]. To reduce noise, we next removed voxels with low temporal signal-to-noise ratio, excluding the lower 50%, and we linearly detrended the time series of each voxel followed by *Z*-scoring. We then median-averaged all functional volumes corresponding to the same trial, obtaining one multivoxel pattern per trial, which served as the basis for MVPA. Specifically, we computed pairwise Spearman correlations between all trials of a given category (e.g., places vs places) as well as across categories (e.g., places vs objects and people). These comparisons were cross-validated across runs to avoid effects of autocorrelated time series noise (i.e., we excluded comparisons of trials that were tested in the same run). If eye movements were more similar between trials of the same category than between trials of different categories, this should result in overall higher Spearman correlations for the within-category comparisons compared with the across-category comparisons. We tested this possibility using one-tailed paired *t* tests. In addition, because averaging across volumes may reduce sensitivity to eye-movement effects within each trial, we further repeated these analyses for each of the four individual volumes acquired in each trial.

#### Linear mixed-effect analysis

To look at the whole-brain memory effects, we employed a linear mixed-effect model (3dLME) in each hemisphere separately. The model comprised a single factor: category (people, places, objects).

#### Statistical approach

ROI statistical analyses were performed using the RStudio software package (version 1.1). For all analyses we conducted repeated-measure analysis of variance (rmANOVA). When Mauchley's test of sphericity was violated, main effects and interactions were corrected using the Greenhouse–Geisser (GG) correction to allow appropriate interpretation. When a significant three-way interaction was observed, we performed two separate two-way ANOVAs to explore the nature of the interaction. When a significant two-way interaction was observed, post hoc paired *t* tests were conducted and corrected for multiple comparisons using Bonferroni’s correction.

## Results

Our goals here were twofold. First, we aimed to replicate prior findings of distinct regions in MPC for the recall of people and places. Second, we explored whether any additional region could be identified via selective recruitment during the recall of personally familiar objects. Prior work (Silson et al., 2019b) highlighted the functional link between the medial–lateral axis of VTC and the ventral–dorsal axis of MPC for perceptual and mnemonic representations of people and places. Here, we extended this work to include recall of personally familiar objects to see if (1) recall of such items recruited a spatially distinct area of MPC and (2), if so, whether this area was located in between the place and people regions as predicted based on the functional organization of VTC (Fig. 1*A*,*B*). Below we focus initially on our whole-brain and a priori ROI analyses within MPC itself, before exploring memory-recall effects outside of MPC at both the whole-brain level and within our post hoc ROIs.

### Replication of people and place recall within MPC

**Figure 2.**
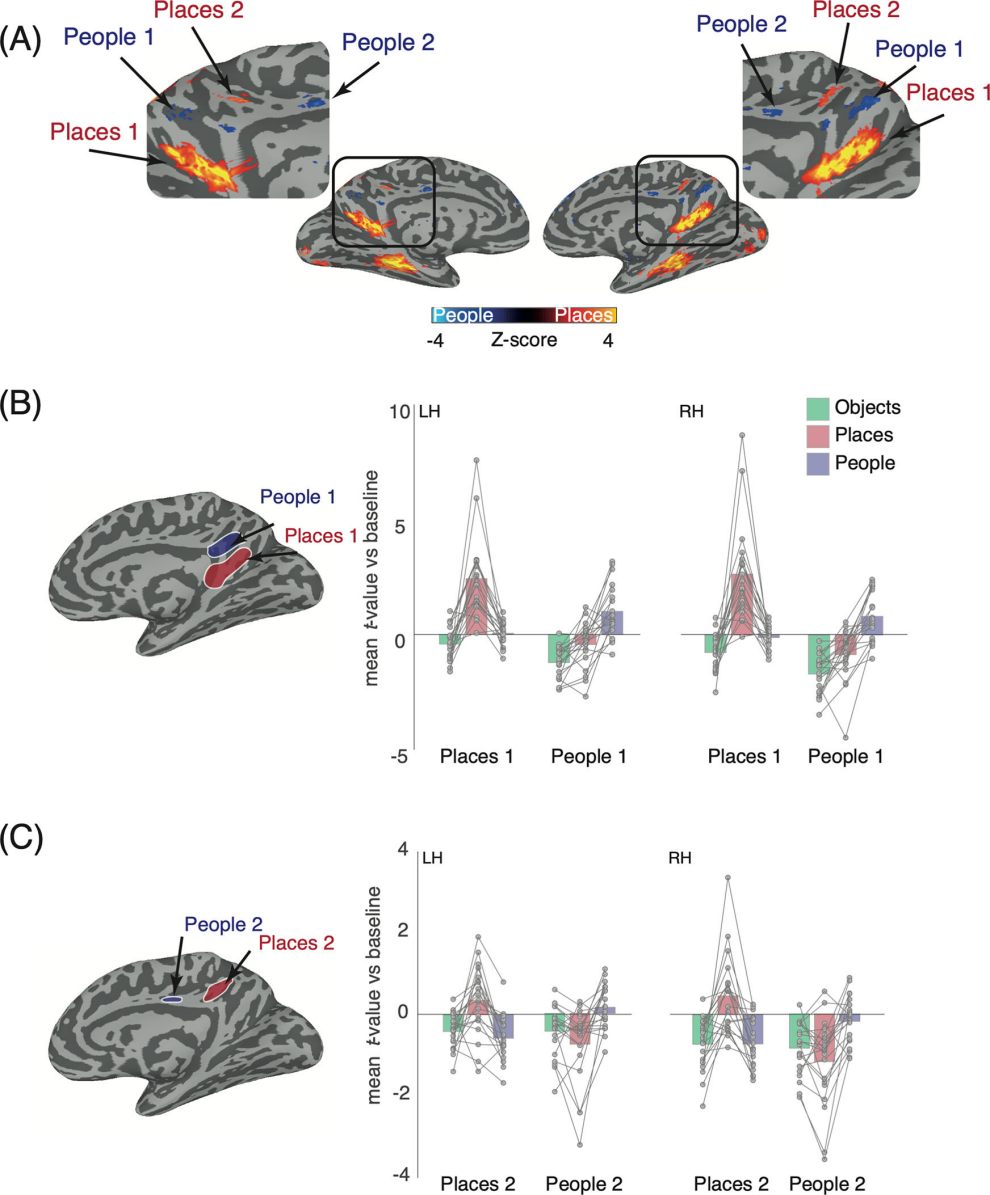
Recall effects in MPC and a priori ROIs. ***A***, Partially inflated medial views of both hemispheres are shown. The contrast of people versus places (*p* = 6.3^−5^; nodewise *q* = 9.2^−4^; corrected for multiple comparisons) is overlaid with cold colors representing regions of MPC recruited during people recall and hot colors representing regions of MPC recruited during place recall. Consistent with prior work, an alternating pattern of people and place recall is evident throughout MPC. Enlarged versions of MPC are inset and demonstrate the alternating pattern. ***B***, A partially inflated medial view of the right hemisphere is shown with a priori Places 1 (blue) and People 1 (red) ROIs overlaid. Bars represent the mean group averaged response to each category (objects, green; places, red; people, blue) in both hemispheres. Individual participant data points are plotted and connected. In both hemispheres, recall of places elicited the largest response in Places 1 with responses during people or object recall being close to or below zero. In People 1, responses were maximally positive during people recall but negative during both object and place recall in both hemispheres. ***C***, Same as ***B*** but for Places 2 and People 2. Overall, a similar pattern of results is evident, although smaller in magnitude. Place recall maximally drove responses in Places 2 (bilaterally), whereas people recall maximally drove responses in People 2 (bilaterally).

Figure 2*A* depicts the whole-brain contrast of places versus people (*p* = 6.3^−5^; nodewise *q* = 9.24) across medial views of both hemispheres, with MPC enlarged. An alternating pattern of people and place recall was present throughout MPC in both hemispheres. Indeed, the topography of these regions closely matches those reported in prior work despite originating from an independent group of participants. To explore these data further, we calculated the mean response (*t* value vs baseline) to each category within a priori ROIs (People 1, Places 1, People 2, and Places 2; Fig. 2*B*), which we split into two sets: a larger more ventral pair (i.e., People 1, Places 1) and a smaller more dorsal pair (i.e., People 2, Places 2), based on prior work (Silson et al., 2019b). A consistent pattern was observed within the a priori ROIs, and we discuss each pair of regions in turn.

First, recall of places selectively recruited Places 1 and the recall of people selectively recruited People 1 bilaterally (Fig. 2*B*). Interestingly, the recall of objects produced negative responses in both regions but to varying degrees with responses more negative during object recall in People 1. To quantify these effects, we conducted a three-way rmANOVA with hemisphere (left, right), ROI (Places1, People1), and category (objects, places, people) as within-participant factors. The main effects of hemisphere (*F*_(1,19)_ = 10.21; *p* = 0.004), ROI (*F*_(1,19)_ = 25.79; *p* = 6.665), and Category (*F*_(2,38)_ = 29.74; *p* = 1.68^−8^) were all significant, reflecting on average larger responses in the right hemisphere, in Places 1 and for the recall of places, respectively. These were qualified however by significant interactions of hemisphere × ROI (*F*_(1,19)_ = 6.16; *p* = 0.02), hemisphere × category (*F*_(2,38)_ = 3.74; *p* = 0.03), ROI × category (*F*_(2,38)_ = 42.66; *p* = 3.93^−8^, GG-corrected), and hemisphere × ROI × category (*F*_(2,38)_ = 5.24, 0.009).

To better interpret the three-way interaction, we performed two two-way rmANOVAs in each hemisphere separately. In the left hemisphere, the main effects of ROI (*F*_(1,19)_ = 20.21; *p* = 0.0002) and category (*F*_(2,38)_ = 29.26; *p* = 2.02^−8^) were significant, again reflecting larger responses in Places 1 over People 1 and for the recall of places over either people or objects. Importantly, these main effects were qualified by a significant ROI × category interaction (*F*_(2,38)_ = 34.38; *p* = 1.37^−7^, GG-corrected), reflecting the differential pattern of responses across the two ROIs. In the right hemisphere, the main effects of ROI (*F*_(1,19)_ = 27.95; *p* = 4.19^−5^) and category (*F*_(2,38)_ = 28.07; *p* = 8.59^−7^, GG-corrected) were significant (larger responses in Places 1 and for places over people or objects) and were qualified by a significant ROI × category interaction (*F*_(2,38)_ = 47.46; *p* = 2.36^−8^, GG-corrected), reflecting the differential pattern of responses across the two ROIs. A series of paired *t* tests (Bonferroni corrected, *α* = 0.004) confirmed greater responses in Places 1 bilaterally during place recall as compared with either people (LH, *t*_(19)_ = 5.89; *p* = 1.12^−5^; RH, *t*_(19)_ = 5.84; *p* = 1.25^−5^) or objects (LH, *t*_(19)_ = 5.99; *p* = 8.99^−6^); RH, *t*_(19)_ = 6.01; *p* = 8.73^−6^). The response to people was also different to objects in the right hemisphere (*t*_(19)_ = 3.28; *p* = 0.003), but not the left (*t*_(19)_ = 2.30; *p* = 0.03). In People 1, responses during people recall were greater than both places (LH, *t*_(19)_ = 4.63; *p* = 0.001; RH, *t*_(19)_ = 6.31; *p* = 4.60^−6^) and objects (LH, *t*_(19)_ = 6.64; *p* = 2.34^−6^; RH, *t*_(19)_ = 7.87; *p* = 2.11^−7^). The response to places was greater than the response to objects in the left hemisphere (*t*_(19)_ = 3.74; *p* = 0.001), but not the right (*t*_(19)_ = 2.99; *p* = 0.007).

Second, we replicated a striking finding of previous work: another more dorsal pair of regions also appear to be selectively recruited during people and place recall (i.e., People 2, Places 2). Here, like previously, selective responses were observed in both ROIs despite lower overall magnitudes relative to their larger, more ventral counterparts. That is, recall of places still maximally drove positive responses in Places 2, whereas recall of people resulted in the most positive responses in People 2. Again, responses during object recall were almost always negative across both ROIs (Fig. 2*C*). Consistent with the analyses described above, we again conducted a three-way rmANOVA, revealing main effects of hemisphere (*F*_(1,19)_ = 10.09; *p* = 0.004) and category (*F*_(2,38)_ = 3.37; *p* = 0.04), reflecting on average larger responses in the right hemisphere and in People 2. There was no main effect of ROI (*F*_(1,19)_ = 3.28; *p* = 0.08). Only the ROI × category interaction (*F*_(2,38)_ = 30.94; *p* = 5.29^−7^, GG-corrected) was significant (*p* > 0.05, in all other cases). Given that hemisphere did not interact with the other factors, we collapsed across hemispheres and ran a two-way rmANOVA. The main effect of ROI (*F*_(1,19)_ = 3.28; *p* = 0.08) was not significant, but the main effect of category (*F*_(2,38)_ = 3.37; *p* = 0.04) was, as was the ROI × category interaction (*F*_(2,38)_ = 20.94; *p* = 5.29^−7^, GG-corrected). Again, a series of paired *t* tests (Bonferroni’s corrected, *α* = 0.008) revealed greater responses during place recall in Places 2 than either people (*t*_(19)_ = 5.72; *p* = 1.63^−5^) or objects (*t*_(19)_ = 4.48; *p* = 0.0002). The response to people was not different to objects (*t*_(19)_ = 0.51; *p* = 0.61). In People 2, people recall was greater than recall of both places (*t*_(19)_ = 4.33; *p* = 0.0003) and objects (*t*_(19)_ = 3.61; *p* = 0.001). The recall of places was not different from objects (*t*_(19)_ = 1.91; *p* = 0.07).

### No evidence for object recall within MPC

**Figure 3.**
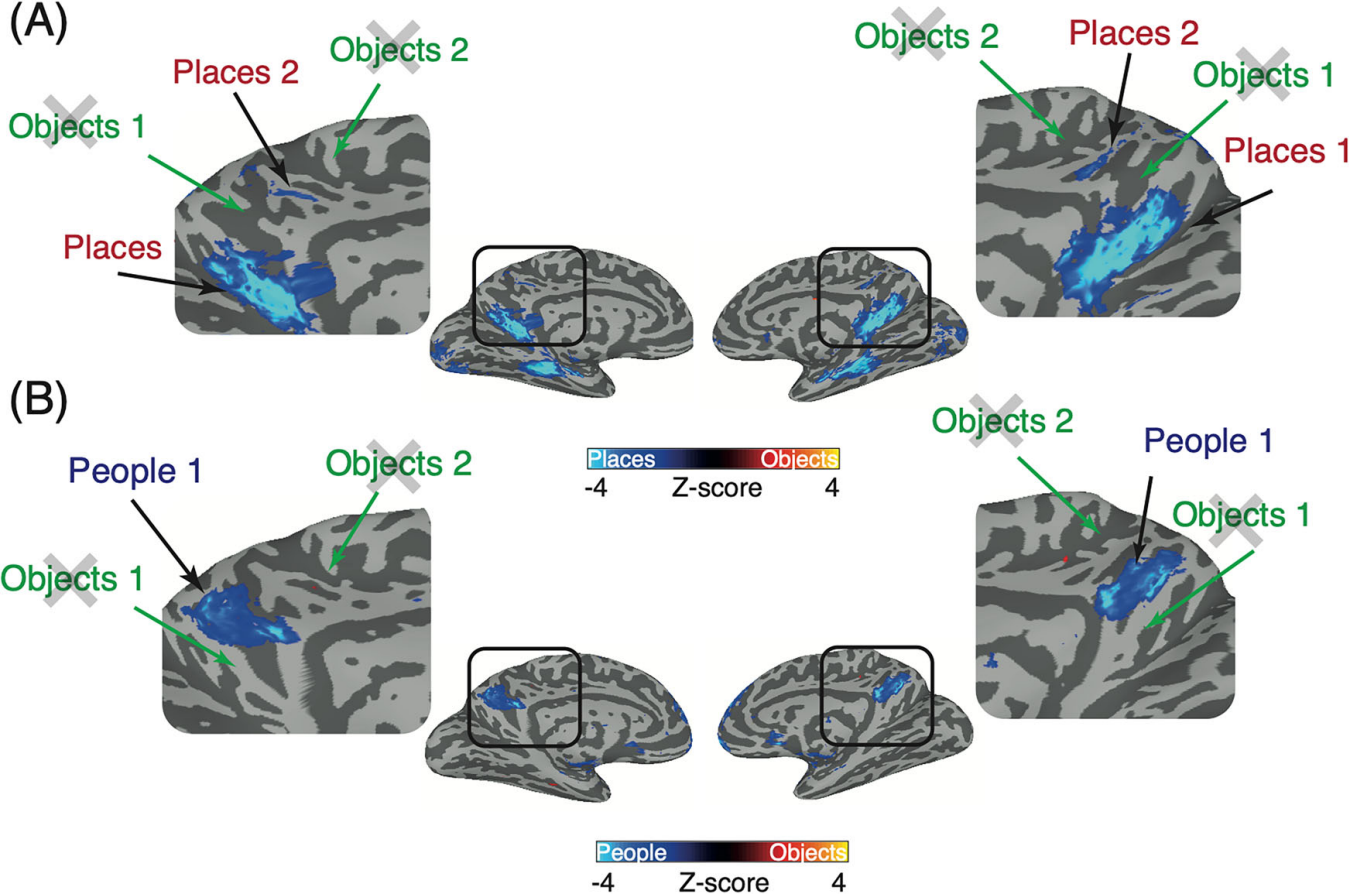
Lack of object recall within MPC. ***A***, Partially inflated medial views of both hemispheres are shown. The contrast of places versus objects (*p* = 6.3^−5^; nodewise *q* = 9.2^−4^, corrected for multiple comparisons) is overlaid with cold colors representing regions of MPC recruited during place recall and hot colors representing regions of MPC recruited during object recall. No suprathreshold object clusters are present in the predicted locations, whereas place-recall clusters are easily identifiable. ***B***, Same as ***A*** but for the contrast of people versus objects. Again, there is a lack of object recall but clear people recall.

**Figure 4.**
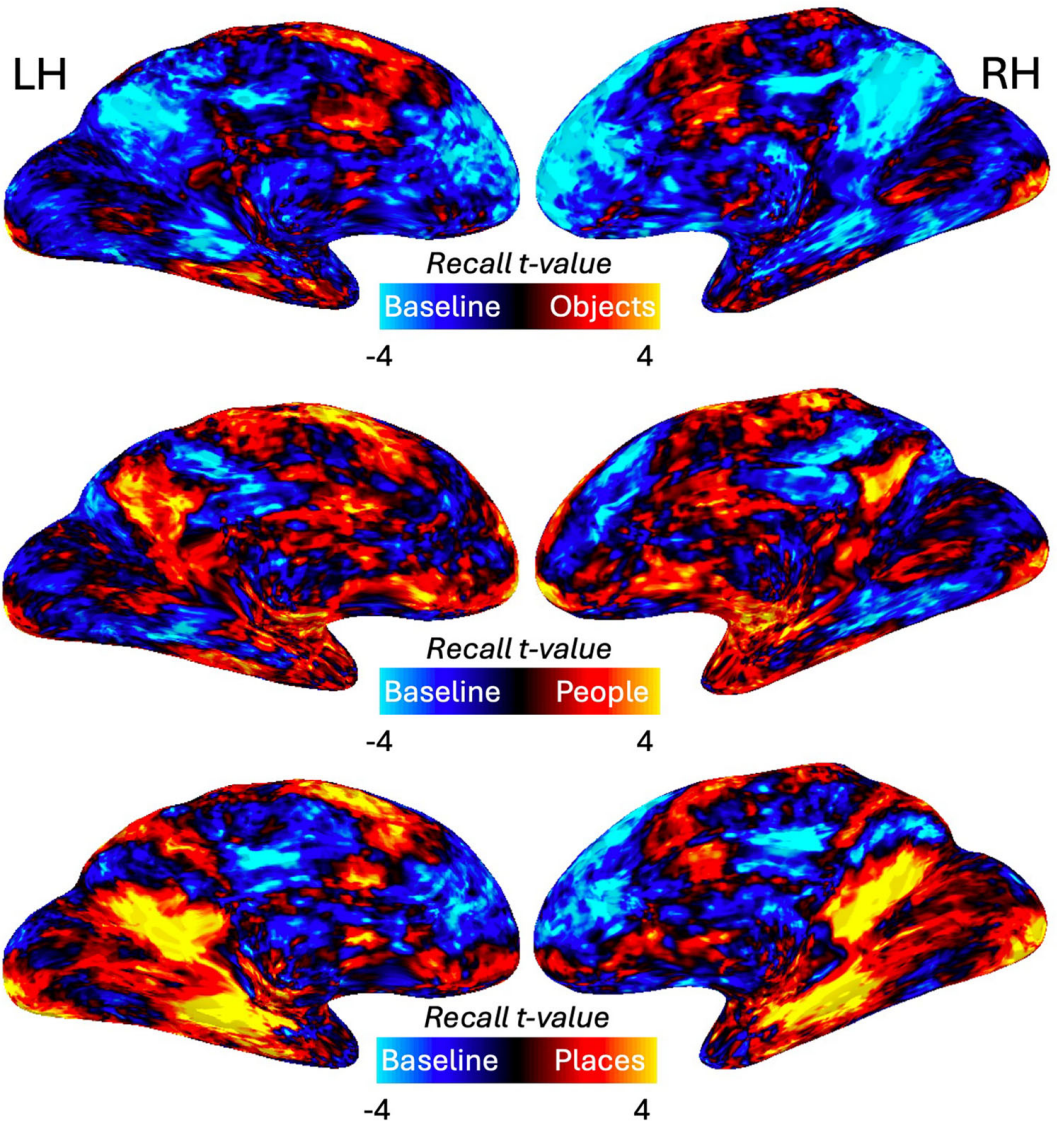
Object, people, and place recall within MPC versus baseline. Top, Medial views of the left and right hemispheres are shown with the group-average response during object recall (hot colors) versus baseline (cold colors). Within MPC there is little to no object-related recall activity. Middle, Same as the top but for people versus baseline. Here, portions of MPC show robust responses during people recall. Bottom, Same as the top but for places versus baseline. Different portions of MPC show robust responses during place recall.

Having replicated prior work on people and place recall, we next examined object recall. Based on the topography of categorical preferences along the medial–lateral axis of VTC (Fig. 1*B*), we predicted that object recall would also recruit distinct region(s) of MPC in between those recruited during people and place recall. However, there was a striking lack of object-related activity within MPC. Indeed, when contrasting object recall directly with either places (Fig. 3*A*) or people (Fig. 3*B*), no suprathreshold object-recall clusters are evident, whereas clusters recruited during place and people recall are easily identifiable and closely match those highlighted in Figure 2*A*. The lack of object-recall activity within MPC was even evident when comparing objects versus baseline (Fig. 4).

**Figure 5.**
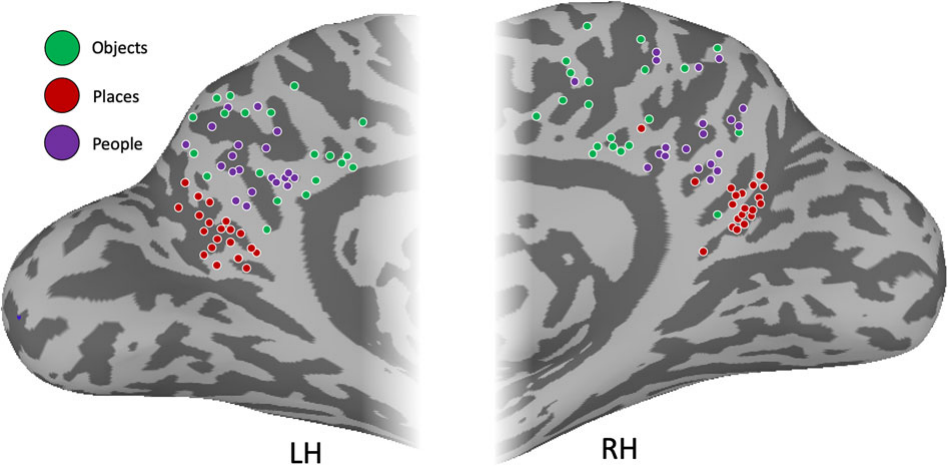
Peak location of place, people, and object recall within MPC. Medial views of the left and right hemispheres are shown with the peak location of place recall (purple), people recall (red), and object recall (green) overlaid. In both hemispheres, place and people recall are clustered along a posterior–anterior trajectory, whereas object recall is far more distributed throughout MPC.

At the group level, there was little to no evidence for object-related recall activity within MPC either when contrasted against people or places or against the baseline period. As a potential explanation for this lack of effect at the group level, we examined the spatial location within MPC of the peak response to each category during recall in each individual participant (Fig. 5). Consistent with prior work, we observed that the peaks of place and people recall were not only spatially clustered but fell largely along a posterior–anterior trajectory with the peaks for place recall being posterior of those for people recall. In contrast, the peaks of object recall were widely distributed throughout MPC, which likely underpins our inability to identify contiguous responses at the group level. Having characterized category-recall responses within MPC, we next asked whether regions outside of MPC also showed category-selective recall responses.

### Distinct category-recall effects outside of MPC

**Figure 6.**
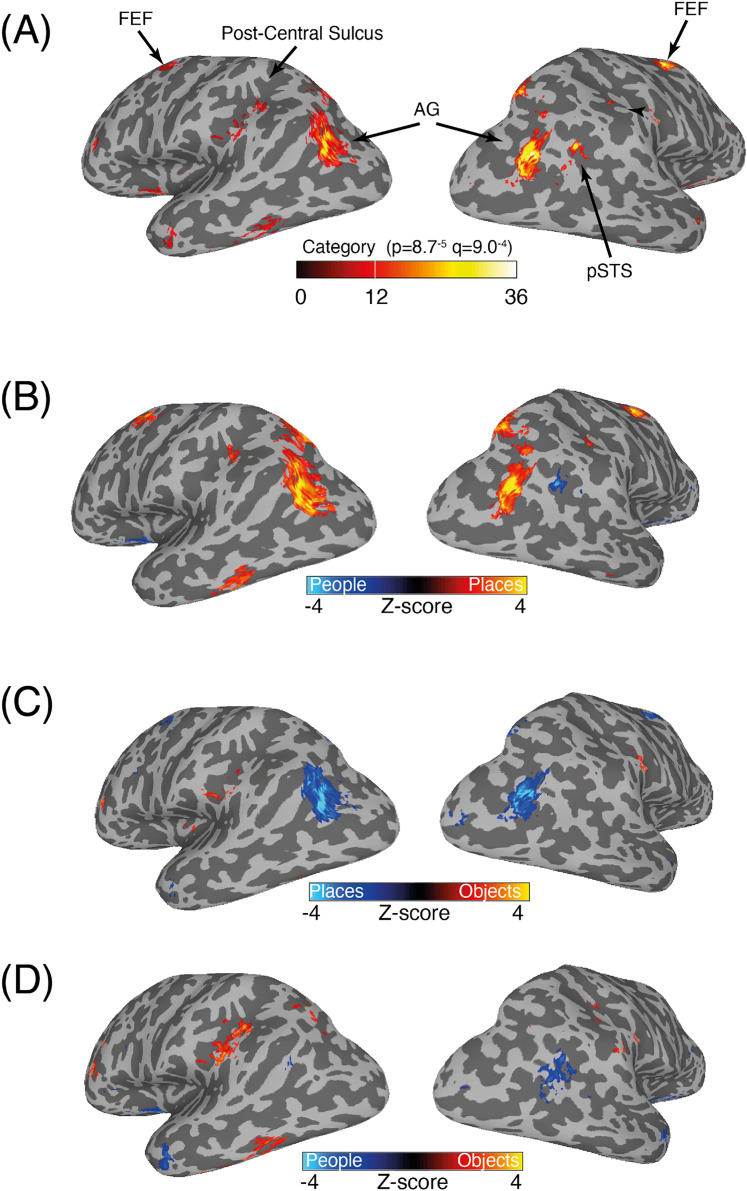
Recall effects outside MPC. ***A***, Partially inflated lateral views of both hemispheres are shown. The main effect of category (*p* = 8.7^−5^; nodewise *q* = 9.0^−4^, corrected for multiple comparisons) is overlaid in false color (*F* statistic for the main effect). Memory-recall effects are present within posterior of the postcentral sulcus, within the AG and putative FEF bilaterally and within pSTS of the right hemisphere. ***B***, The contrast of people versus places (*p* = 6.3^−5^; nodewise *q* = 9.2^−4^) is overlaid onto the same medial views as ***A***. Both the AG and FEF were recruited during place recall, whereas people recall recruited pSTS in the right hemisphere. ***C***, Same as ***B*** but for the contrast of places versus objects. Object-recall effects were evident in regions inferior and posterior to the primary somatosensory cortex and in the vicinity of AIP. Place-recall effects remained in the AG and FEF. ***D***, Same as ***B*** but for the contrast of people versus objects. Again, object-recall effects were evident in the SC, particularly in the left hemisphere. People-related recall in right pSTS also remained.

Prior work (Gilmore et al., 2015; Peer et al., 2015; Favila et al., 2019; Silson et al., 2019a,b; Bainbridge et al., 2021; Steel et al., 2021) reported that memory-recall effects were not restricted to MPC and could also be found on both the lateral surface of the occipitotemporal cortex and in frontal regions. Figure 6*A* depicts the whole-brain effect of category on lateral views of both hemispheres (*p* = 8.7^−5^; nodewise *q* = 9.0^−4^). Significant effects are present near the postcentral sulcus of the left hemisphere, in posterior angular gyrus (bilaterally), the pSTS in the right hemisphere, and the dorsal frontal lobe (bilaterally), likely corresponding to the FEF. To examine these effects in more detail, we adopted the same approach taken above and next explored the category contrasts. Figure 6*B* depicts the contrast of places versus people across the same lateral views. Lateral and frontal memory-recall responses are dominated by place recall, in particular in the angular gyrus and the (putative) FEF. A small significant cluster of people-related recall was identified within the pSTS in the right hemisphere only. Figure 6, *C* and *D*, depicts the contrast of object versus places and objects versus people across the same lateral views. Unlike MPC, we did observe significant object-related clusters here, particularly within inferior and posterior portions of the postcentral sulcus in the left hemisphere in the vicinity of the supramarginal gyrus (particularly prominent for the objects vs people contrast). In the following sections, we explore these effects further in relation to prior studies, in particular sampling the mean response to each category from post hoc ROIs, corresponding to the face-selective pSTS (see Materials and Methods) and the FEF (Wang et al., 2015).

### People-recall effects in the posterior STS

**Figure 7.**
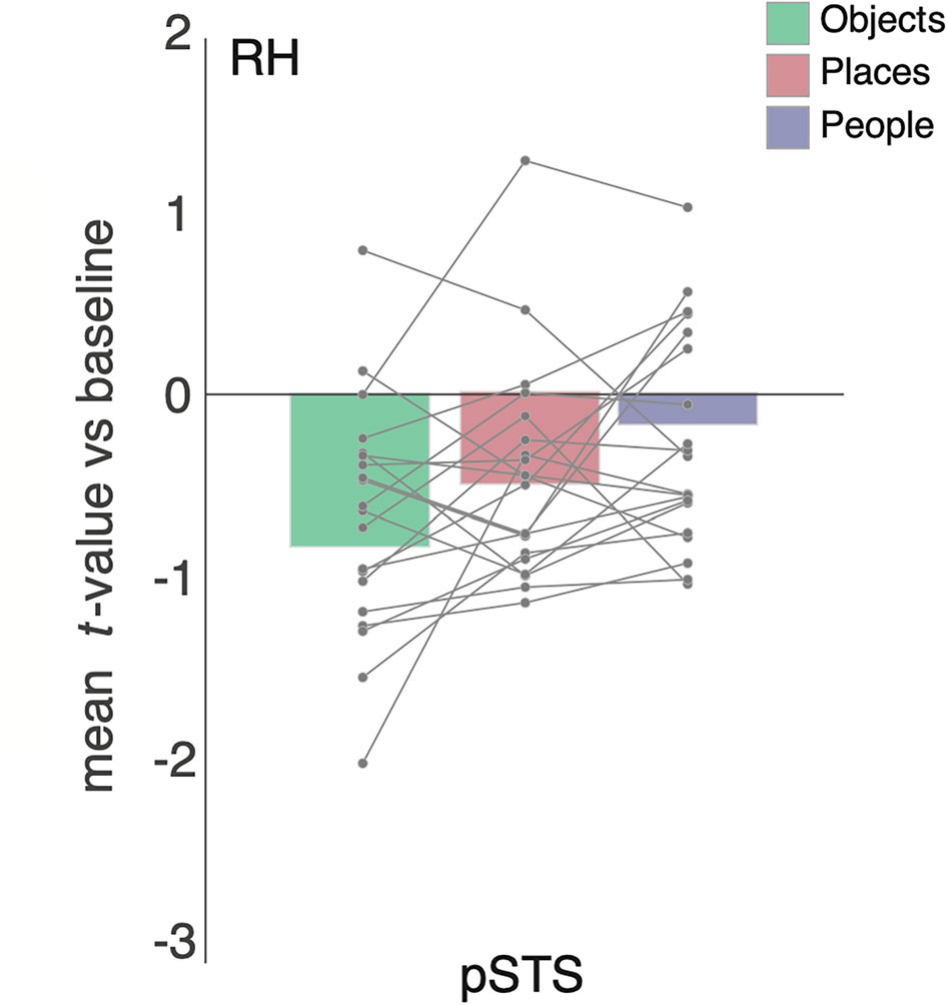
People recall in the right pSTS. Bars represent the mean response to all categories from the pSTS in the right hemisphere. Individual participant data points are plotted and connected. Despite negative responses to all categories, these responses differentiate in the basis of category with responses during people recall being less negative (relatively more positive).

A surprising finding of our whole-brain analyses, which we did not observe in our prior study, was a small cluster within the pSTS of the right hemisphere that appeared when we contrasted either people versus places (Fig. 6*B*) or people versus objects (Fig. 6*D*). This cluster might correspond to the visual face selectivity that has previously been reported in pSTS. To better understand the responses we observed, we sampled the mean response to each category from a group-based ROI for face-selective pSTS taken from an independent group of participants (see Materials and Methods). Despite responses in this region being negative for all categories (Fig. 7), responses were less negative during people recall compared with all other categories. A one-way ANOVA with category (same levels as above) as the within-participant factor showed a main effect of category (*F*_(2,38)_ = 4.22; *p* = 0.02). A series of paired *t* tests (Bonferroni corrected, *α* = 0.0167) revealed that responses during people recall were different from those during recall of objects (*t*_(19)_ = 2.55; *p* = 0.01), but not places (*t*_(19)_ = 1.40; *p* = 0.19). Responses were not different between places and objects (*t*_(19)_ = 1.72; *p* = 0.10).

### Place recall recruits the FEF

**Figure 8.**
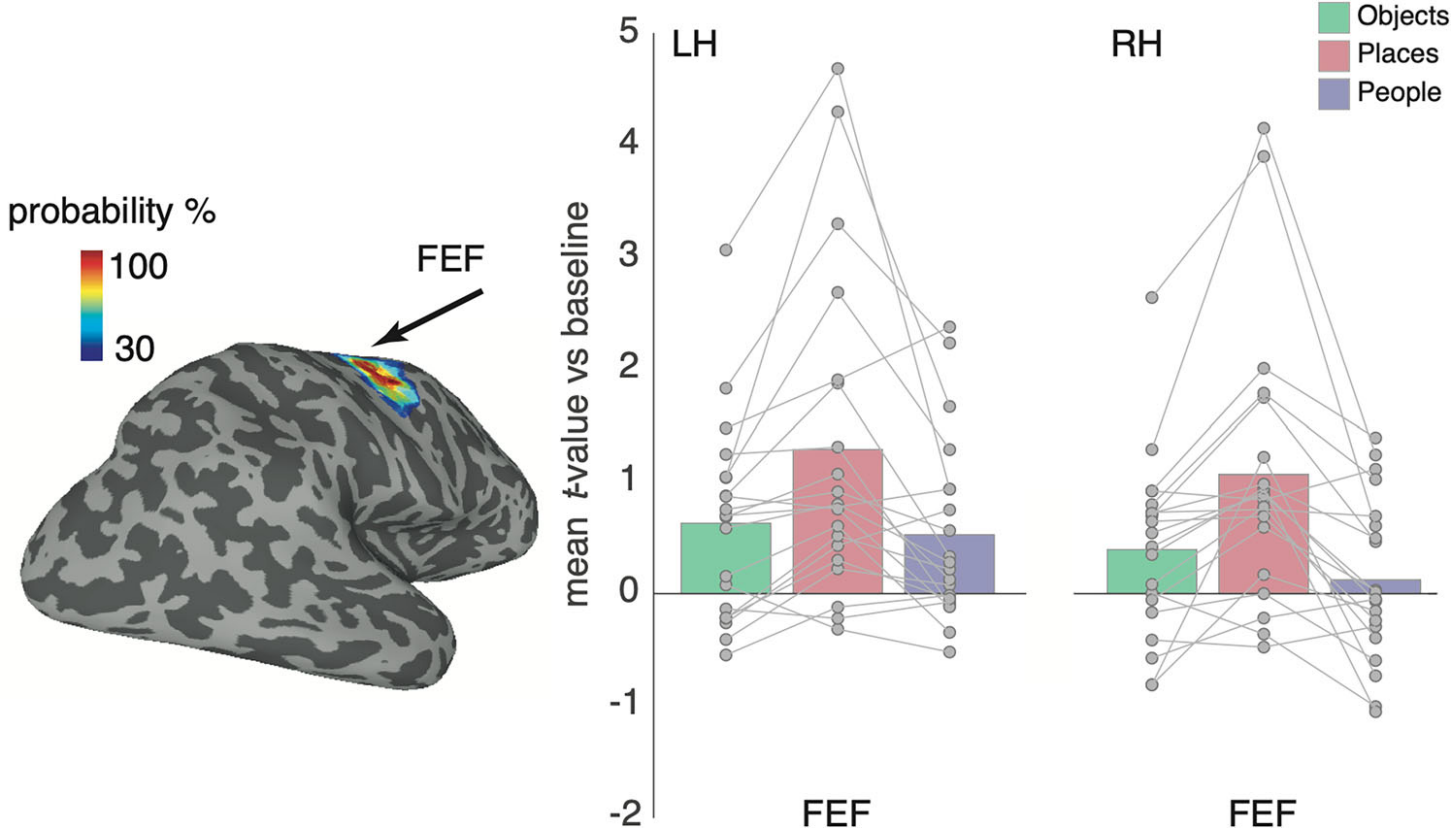
Place recall in the FEF. A lateral view of the right hemisphere is shown with the probabilistic mask for the FEF taken from Wang et al. (2015) thresholded at 30% of the maximum probability. Bars represent the mean response to all categories from this FEF ROI in both hemispheres. Individual participant data points are plotted and connected. Place recall maximally drove responses in both hemispheres. The responses during both people and object recall were weaker but largely equivalent.

Our whole-brain analyses also highlighted significant category effects in the approximate location of the FEF. To quantify this effect and to confirm that it was indeed localized to the FEF, we sampled the mean response to each category from a probabilistic retinotopic FEF mask (Wang et al., 2015) in both hemispheres (Fig. 8). A two-way rmANOVA with hemisphere and category as factors (same levels as above) revealed main effects of hemisphere (*F*_(1,19)_ = 17.41; *p* = 0.0005) and category (*F*_(2,28)_ = 11.84; *p* = 0.0001). On average, the magnitude was larger in the left hemisphere, and we observed larger responses during place recall compared with other categories, respectively). The hemisphere × category interaction (*F*_(2,38)_ = 2.09; *p* = 0.13) was not significant. A series of paired *t* tests (Bonferroni corrected, *α* = 0.008) revealed that responses during place recall were greater in the FEF (bilaterally) than either recall of people (LH, *t*_(19)_ = 3.27; *p* = 0.003; RH, *t*_(19)_ = 4.37; *p* = 0.0003) or objects (LH, *t*_(19)_ = 3.54; *p* = 0.002; RH, *t*_(19)_ = 3.90; *p* = 0.0009). Importantly, responses were not different between people and object recall in either hemisphere (LH, *t*_(19)_ = 0.58; *p* = 0.56; RH, *t*_(19)_ = 1.81; *p* = 0.08).

### Object-recall effects in the posterior parietal cortex

**Figure 9.**
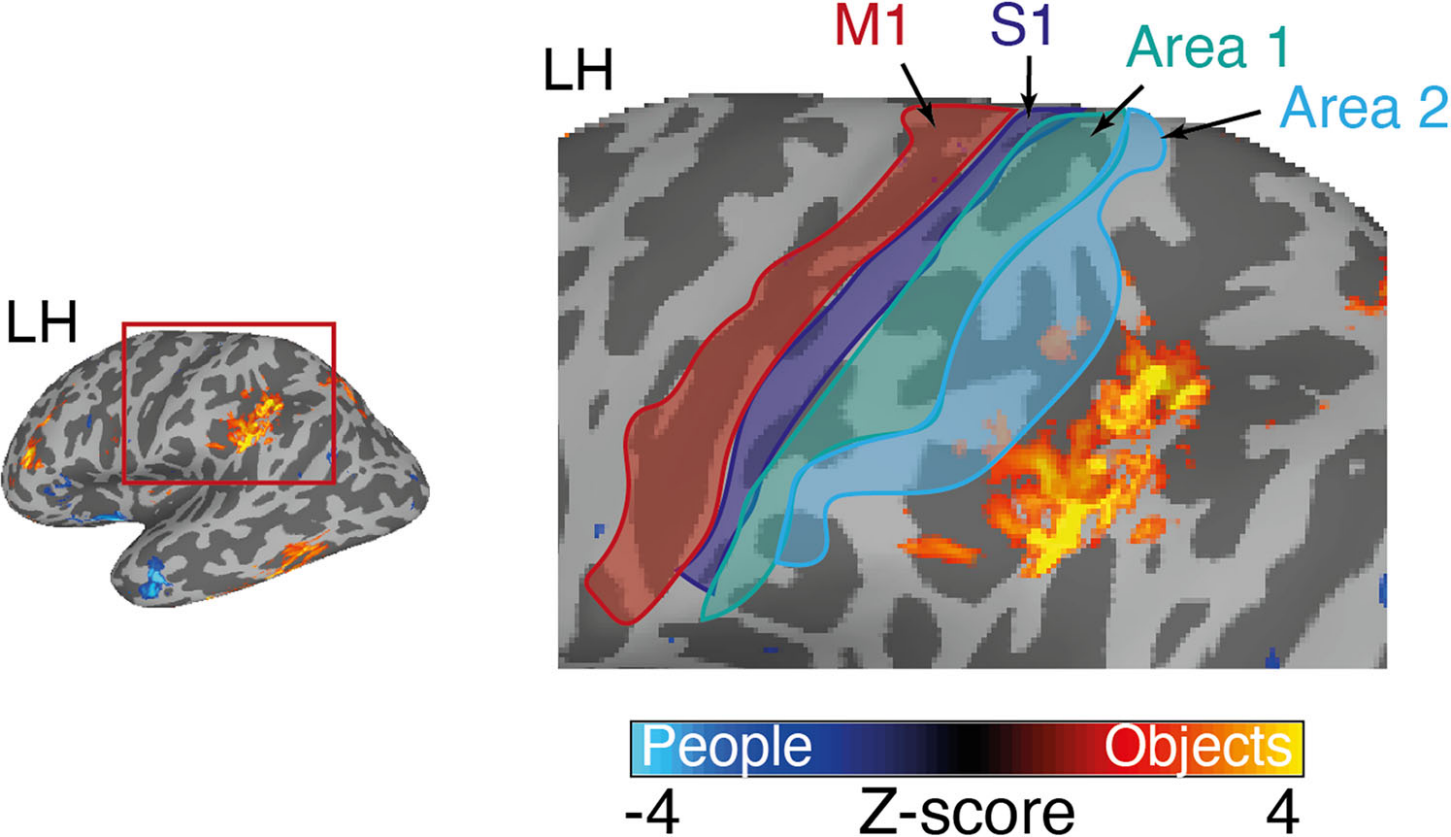
Object recall in the posterior parietal cortex. A lateral view of the left hemisphere is shown with the posterior parietal cortex highlighted. The contrast of people versus objects is overlaid and enlarged to the right. This object recall is in the vicinity of the supramarginal gyrus but is posterior of the primary motor cortex (M1, red outline), primary somatosensory cortex (S1, blue outline), Area 1 (green outline), and Area 2 (cyan outline) taken from a publicly available parcellation (Glasser et al., 2016).

We also found significant category effects in the posterior parietal cortex, in and around the post central sulcus, that were particularly prominent for the contrast of people versus object recall (Fig. 9). This region is posterior to primary motor (M1) and somatosensory (S1) cortices as well as BA 1 and 2 and superior to the secondary somatosensory cortex. Prior work has reported responses in this area associated with grasping, but not reaching (Frey et al., 2005; Konen et al., 2013) or touching (Cavina-Pratesi et al., 2010) and with the visual presentation of tools and other small manipulable objects (Chao and Martin, 2000).

### Recall effects in EVC

**Figure 10.**
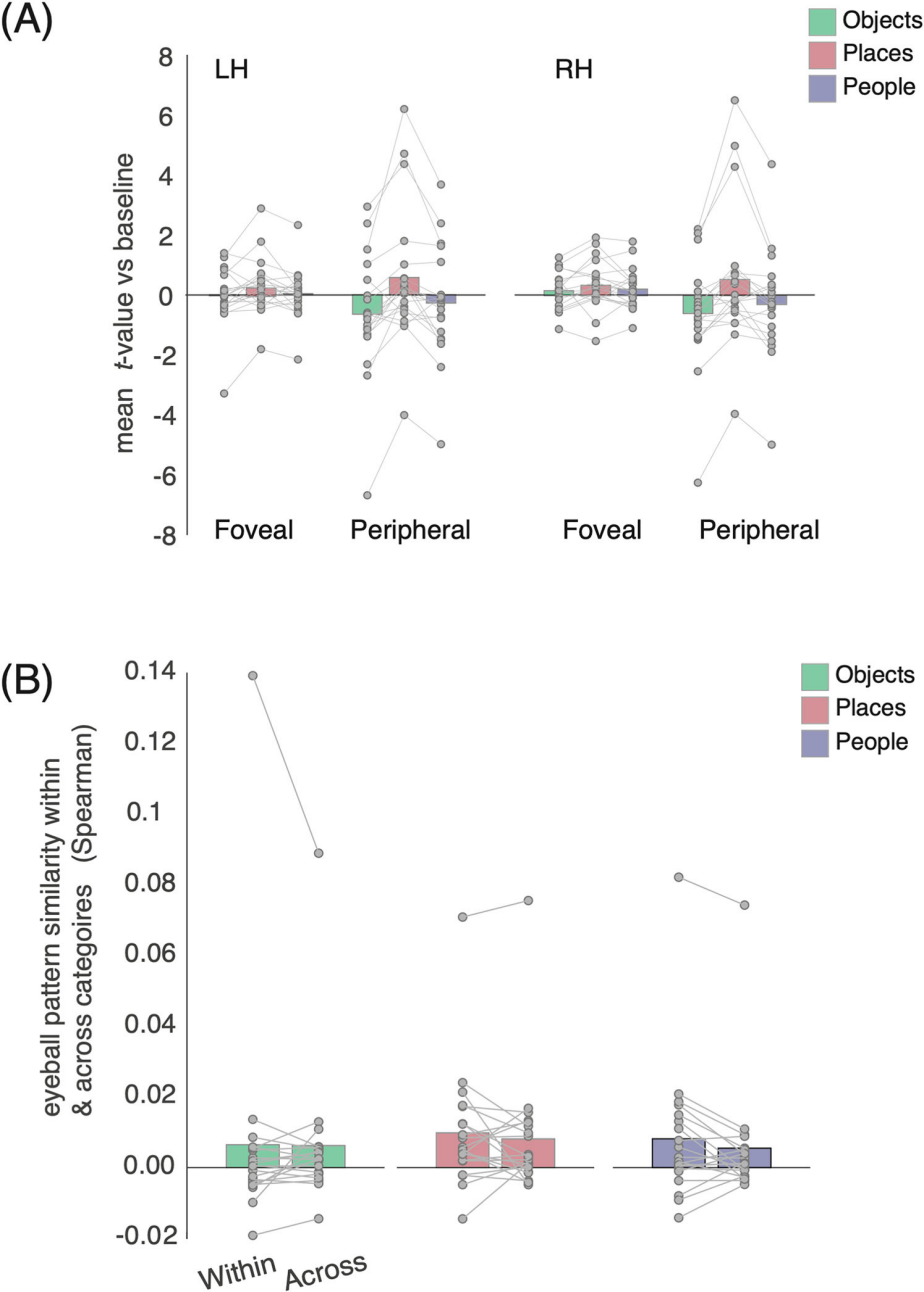
Recall effects in EVC and eyeball pattern similarity within and across categories. ***A***, Bars represent the mean response to all categories from foveal and peripheral EVC in both hemispheres. Overall, responses are largest during place recall in both foveal and peripheral EVC, with larger responses collectively in peripheral EVC. Individual participant data points are plotted and connected. ***B***, Bars represent the mean pattern similarity (Spearman) taken from the eyeballs both within and across categories. Individual participant data points are plotted and connected. In each case, there were no significant differences between the pattern similarity within and across categories.

The contrast of people versus places (Fig. 2) and our whole-brain analysis also hinted at recall effects within EVC (V1–V3). Given the links between foveal/peripheral vision and face/scene processing, we explored potential recall effects in foveal/peripheral EVC using population receptive field data from an independent group of participants (Fig. 10*A*). Accordingly, these data were submitted to a three-way rmANOVA with hemisphere, ROI (foveal, peripheral) and category as within-participant factors. Only the main effect of category (*F*_(38)_ = 38.35; *p* = 0.0001) and the ROI × category interaction (*F*_(38)_ = 13.14; *p* = 0.00004) were significant (*p* > 0.05, in all other cases). Given the nonsignificant effect of hemisphere, these data were collapsed across hemisphere and resubmitted to a two-way rmANOVA with category and ROI as factors. The main effect of ROI (*F*_(19)_ = 0.89; *p* = 0.35) was not significant, but both the main effect of category (*F*_(38)_ = 10.75; *p* = 0.0001) and the ROI × category interaction (*F*_(38)_ = 13.18; *p* = 0.00004) were, reflecting on average larger responses within the peripheral ROI and positive responses during place recall but negative responses during both people and object recall. A series of paired *t* tests (Bonferroni corrected, *α* = 0.0083) revealed no differences between conditions in the foveal ROI (*p* > 0.07, in all cases) but in the peripheral ROI responses during place recall were greater than recall of either people (*t*_(19)_ = 3.70; *p* = 0.001) or objects (*t*_(19)_ = 4.41; *p* = 0.0002). There were no differences between the recall of objects and people (*t*_(19)_ = 1.72; *p* = 0.10).

### Eyeball-voxel analysis: an effect of category on eye movements?

Our exploratory whole-brain analysis revealed place-recall–related activity in the FEF, a retinotopically organized region often associated with oculomotor control (Mackey et al., 2017; Coiner et al., 2019). We thus wondered if the observed FEF activity reflected eye movements that participants may have performed during recall. Indeed, prior work suggests that eye movements play a functional role in memory recall (Ryan and Shen, 2020). We reasoned that such recall-related eye movements may be more pronounced for places (or scenes) compared with objects or people, because of the often-complex arrangement and large number of features associated with a given place.

While we did not acquire eye-tracking data during scanning, recent work showed that eye movements affect the multivoxel pattern (MVP) of the eyeballs and that the so inferable eye movements explain activity in the FEF (Frey et al., 2021). Therefore, to test if category-specific patterns in eye movements could explain our FEF results, we tested if the eyeball MVP in our data was indicative of the recalled category. We first extracted the eyeball MVP at each time point following established approaches (Frey et al., 2021) and averaged the MVP across functional volumes within each trial. We then computed the pairwise pattern dissimilarity between all trials of each category (within category) as well as between trials of different categories (across category). If participants moved their eyes more during recall of places compared with objects and people, we expected to find a weaker pattern dissimilarity for the within-category comparisons versus the across-category comparisons. This was not the case (Fig. 10*B*; paired *t* test of within vs across, *p *> 0.13, for all categories). However, each trial was 10 s long, and the effect of eye movements on the MVP may have been lost when averaging across TRs. We therefore repeated the above analysis for each individual functional volume within each trial, again not finding an effect of category. Thus, this indirect measure of eye movements did not show a difference in viewing behavior between categories.

## Discussion

The current study was designed to accomplish two goals. The first was to provide an independent test of the people- and place-recall results reported previously (Silson et al., 2019b). The second was to test whether object recall recruits distinct region(s) of MPC, akin to how perception of objects recruits distinct regions of VTC (i.e., pFS). Replicating our prior work to a striking degree, we identified an alternating pattern of people and place recall throughout MPC. However, despite strong predictions that were based on the similarities between VTC and MPC organization, object-recall effects were not observed within MPC itself. They were however observed in regions posterior and inferior to the primary somatosensory cortex, particularly in the left hemisphere. Additional recall effects were evident within the right pSTS during people recall and the FEF and EVC during place recall.

### Memory-recall effects within MPC

Contrasting the responses elicited during people and place recall recapitulated original findings with a striking level of consistency. Such consistency was present not only within the topography and areal extent of the whole-brain activations but also the pattern of responses within our MPC ROIs. The identification of four regions within MPC differentially recruited during people and place recall strongly supports our original report (Silson et al., 2019b) and recent independent fMRI (Deen and Freiwald, 2021) and neurophysiological evidence (Woolnough et al., 2020).

Within MPC itself, we found no evidence for distinct recruitment during object recall either at the whole-brain or ROI analysis levels, despite strong predictions based on the otherwise parallel functional organization of VTC. Naturally, the absence of an effect is difficult to interpret, and it does not rule out that under other experimental settings, MPC may show object selectivity during recall. In the following, we discuss several factors that could have contributed to this null result. First, recalling personally familiar people (e.g., mother) and places (e.g., childhood home) conceivably evokes stronger semantic, contextual, and emotional associations than the recall of familiar objects (e.g., wallet). It is possible that the strong people and place responses and lack of object recall within MPC reflect these differences in emotional/contextual and/or semantic importance (Bar and Aminoff, 2003). Unfortunately, we did not collect subjective ratings of the emotional/semantic significance of recalled items to test this possibility directly, but taking these and other factors into account is a key goal of our future work. Second, on the one hand, it is possible that our internal representations of objects, particularly the types of objects selected by our participants, (e.g., watch, wallet) are evolutionarily too recent relative to representations of people and places to recruit distinct regions within MPC. Unlike representations of people and places, our species’ experience with these objects comes solely from the last few hundred years (if that). On the other hand, the use of tools and other small manipulable objects encompasses more than two million years of human history. Third, previous work put forward an organizational framework for understanding MPC that reflected the medial–lateral axis of VTC (Silson et al., 2019b). Although several different dimensions are thought to be represented along this axis, including eccentricity (Levy et al., 2001; Hasson et al., 2002), animacy (Konkle and Caramazza, 2013), and real-world size (Konkle and Oliva, 2012), categorical preference is arguably the strongest and most robust organization, being present even in infancy (Deen et al., 2017; Kosakowski et al., 2021). Within this organization, responses to objects are generally weaker than those elicited by either faces or scenes. Taken from the perspective of a functional link between VTC and MPC, it is possible that the absence of object-recall effects within MPC reflects this reduced responsiveness in VTC relative to people and places. Along these lines, it is possible that MPC represents the most salient distinctions within VTC only, which might facilitate the accurate recollection of information during memory. It is well known that contrasting faces and scenes produces an antagonistic response within FFA and PPA, respectively. Indeed, across the commonly tested visual object categories, faces reliably produce the largest positive response in FFA but simultaneously the weakest response in PPA and vice versa. Such a salient distinction in VTC could therefore be reflected in the prominence of people and place recall reported here and previously within MPC (Silson et al., 2019b; Woolnough et al., 2020; Bainbridge et al., 2021; Deen and Freiwald, 2021; Steel et al., 2021).

### FEF, place recall, and the role of eye movements

Interestingly, we observed stronger recall activity in the FEF for places compared with people or objects, which we reasoned may reflect a difference in viewing behavior between these categories. This would be in line with the proposed role of the FEF in oculomotor control (Mackey et al., 2017; Coiner et al., 2019) and with the notion that eye movements support memory recall (Ryan and Shen, 2020). While our imaging results predicted that participants performed more eye movements when recalling places compared with the other categories, we did not have concurrent eye-tracking data to test this prediction directly. However, prior work showed that eye movements affect the voxel pattern of the eyeballs (Frey et al., 2021). We therefore used representational similarity analysis to test if the eyeball voxels carried information about category. While this was not the case in our data, we would like to emphasize that there are multiple potential reasons why. An obvious one is that there might not have been a difference in viewing behavior between categories. However, our task, data, and analysis measures were also not optimal to find these effects even if they were present. First, the word cue remained on screen for the entire duration of the trial, meaning that one would expect a certain amount of eye movements in all trials irrespective of category. The effects we were looking for were therefore likely very small. Second, our repetition time of 2.5 s may have simply been too long for picking up these small effects, because eye movements occur at a much higher rate. Even if there was an effect of category, it may have averaged out on the level of the functional volumes. Given these possible explanations, we feel it would be wrong to conclude that viewing behavior does not contribute to our results and the possible link between category-specific recall, FEF activity, and eye movements remains a key question for future work. For the present work, we conclude that we do not find evidence for an effect of eye movements on our neuroimaging results.

### People recall in right pSTS

Importantly, we identified people-recall effects within pSTS in the right hemisphere, which we did not observe in our original report (Silson et al., 2019b). Using an independent group-based ROI for the face-selective pSTS, we found that responses were heavily negative during both place and object recall in pSTS, whereas during people-recall responses were close to zero on average. Importantly, the current study only used personally familiar stimuli during recall, whereas our prior work collapsed across both famous and personally familiar effects when contrasting people versus place recall. The previously reported advantage for recall of personally familiar over famous items potentially explains why the pSTS was identified in the current study and not our prior work. The fact that responses were less negative for the preferred category is reminiscent of the negative responses during visual perception of scenes and faces in MPC reported previously (Silson et al., 2019b).

### Object-recall effects

Interestingly, our whole-brain analyses highlighted category-recall effects inferior and posterior to the primary somatosensory cortex that were particularly prominent when contrasting objects versus people. This object-recall response falls in the vicinity of the supramarginal gyrus and postcentral sulcus, which have been implicated in the representation of tools and grasping behavior (Johnson-Frey et al., 2005; McDowell et al., 2018). The majority of recalled objects in this study were small, graspable, and manipulable. It is possible that recall of such items automatically generated associated motor representations, similar to what has been reported previously during the visual perception of familiar manipulable objects (Smith and Goodale, 2015). This interpretation is in line with models that suggest the organization of category representations in the brain is grounded in action (and perceptual/emotional) systems (Martin, 2016).

### Recall effects in EVC

At first glance, the fact that recall of places recruited peripheral portions of EVC to a greater extent than the recall of either people or objects is consistent with the association of scenes and faces/objects with peripheral and foveal visual processing, respectively. However, the increased peripheral activity during place recall could also reflect the fact that on average the place cues tended to contain almost twice as many characters and thus extend more into the periphery. Future studies in which the target word was presented briefly and then removed during recall could explore this effect more definitively as well as be better placed to assess any categorical effects in eye movements during recall.

## Conclusions

Categorical memory-recall effects within MPC appear restricted to those representing either people or places. The prevalence of people and place representations within MPC is consistent not only across studies from our group (Silson et al., 2019b; Bainbridge et al., 2021; Steel et al., 2021) but also independent studies using different imaging methodologies (Woolnough et al., 2020; Afzalian and Rajimehr, 2021; Deen and Freiwald, 2021). This contrasts MPC to VTC, possibly because only the most salient categorical representations within VTC (i.e., faces and places) are recapitulated within MPC in the context of memory. While our data provide no evidence for object-recall effects within MPC itself, we did observe such effects within the vicinity of regions implicated in representing tools and grasping. In sum, the functional organization of MPC is not a direct mirror image of the one of VTC, despite clear parallels surrounding the recall of places and people, which we replicated.

## Data Availability

Group-level surface statistical maps can be found at the Open Science Framework page for this project (https://osf.io/8ugqk/).

## Synthesis

Reviewing Editor: Niko Busch, Westfalische Wilhelms-Universitat Munster Decisions are customarily a result of the Reviewing Editor and the peer reviewers coming together and discussing their recommendations until a consensus is reached. When revisions are invited, a fact-based synthesis statement explaining their decision and outlining what is needed to prepare a revision will be listed below. The following reviewer(s) agreed to reveal their identity: NONE. Note: If this manuscript was transferred from JNeurosci and a decision was made to accept the manuscript without peer review, a brief statement to this effect will instead be what is listed below.

This manuscript was transferred from the Journal of Neuroscience, where it was reviewed by three expert reviewers. While the reviews criticized the lack of novelty of the research and noted that several analyses yielded null findings, they also acknowledged that the manuscript is well-written, addresses a relevant research question, and employs sound methods, making it a valuable contribution to eNeuro's mission of promoting rigorous and transparent science, regardless of perceived novelty or whether the findings are positive. The specific comments and requests for revisions mentioned in the reviews were relatively minor, and the authors have successfully addressed these in the revised manuscript submitted to eNeuro.

I am therefore pleased to accept the manuscript for publication in eNeuro without additional external review.

I have only one request: You mention in your letter that you are planning to make the data and code publicly available. I recommend mentioning the repository and placing the corresponding link in the paper so that readers will be aware of it, as we cannot amend this information post-publication.

See for example this paper (under CODE ACCESSIBILITY):

Microsaccades Track Location-Based Object Rehearsal in Visual Working Memory

Eelke de Vries, Freek van Ede

eNeuro 4 January 2024, 11 (1) ENEURO.0276-23.2023; DOI: 10.1523/ENEURO.0276-23.2023

## Author Response

We thank all three reviewers for their constructive comments. Below we provide a point-point response.

Reviewer #1:

Kidder and colleagues document a robust replication of recent studies characterizing distinct functional areas within human MPC relating to people and places, and build on this work to include the addition of objects. Based on previous work in ventral temporal cortex and its connectivity to MPC the authors hypothesize the existence of MPC Object areas, a hypothesis that is logical and very well formulated within the manuscript. This is an interesting study that contributes to the field; however, I believe there are a few issues to be addressed before publication: (1) From the currently presented methods it is unclear exactly how the concept of "personally familiar" is being defined here and how this was articulated to the participants. There is an important distinction between recalling a familiar semantic concept (e.g. a watch) from recalling a specific exemplar of that concept (e.g. the participant's own watch). Based on prior studies (e.g. Silson et al 2019; Woolnough et al 2020; Afzalian and Rejimehr 2021) it appears likely MPC is engaged in memory of known, unique objects while ventral temporal regions are associated with broader semantic concepts. If participants were visualizing a generic watch rather than recalling a specific watch this could be an alternate explanation for the lack of MPC Object activity. The task instructions given to the participants should be detailed further in the methods and the type-token distinction here should be discussed further.

Response: This point is well taken. We agree with the reviewer that prior work, including our own, demonstrates a familiarity bias within MPC. The participant instructions were the same as in our prior work (Silson et al., 2019). Prior to scanning, each participant submitted a list of 12 personally familiar people, places and objects. Thus, if a participant's object list included 'watch' they were instructed to recall their specific watch and not a generic one. We have re-emphasised this point in the methods section of the revised manuscript. (2) It would be helpful to include surface maps of each category against baseline. Currently all the presented plots are of contrasts between conditions. While Objects do not show selective activation over the other categories in MPC it would be informative to see which areas, if any, are engaged during this task.

Response: We thank the reviewer for this suggestion. We have now plotted the unthresholded recall t-values versus baseline for each of the three categories. During object-recall, there are little to no object-related responses within MPC. In contrast, during people and place recall different regions of MPC show robust responses. These images are now included in a revised Figure 2 and shown below for reference.

Revised Figure 4: Object-, people- and place-recall within MPC versus baseline. (Top) Medial views of the left and right hemispheres are shown with the group-average response during object-recall (hot colous) versus baseline (cold colors). Within MPC there is little to no object-related recall activity. (Middle) Same as (Top) but for people versus baseline. Here, portions of MPC show robust responses during people recall. (Bottom) Same as (Top) but for places versus baseline. Different portions of MPC show robust responses during place recall. (3) Relatedly, is there sufficient power to look within individuals to find the point of peak activity for Objects, relative to the peaks for People and Places (e.g. Silson, Steel et al 2019, Figure 5)? This would help confirm an MPC Object area is not being obscured at population-level due to inter-individual differences in localization.

Response: We thank the reviewer for the suggestion. Following the same approach taken previously, we now plot the peak of object-recall relative to the peaks of place and people recall within each participant (Revised Figure 5 and added below for reference). In contrast to the peaks for places and people, which cluster along a posterior-anterior trajectory (as shown previously), the peaks for object-recall are distributed throughout MPC. Such spatial distribution likely underpins both our inability to identify a contiguous region of object-recall within MPC at the group-level and the lack of object-recall responses in our a-priori ROI analysis.

Revised Figure 5: Peak location of place-, people- and object-recall within MPC. Medial views of the left and right hemispheres are shown with the peak location of place recall (purple), people recall (red) and object recall (green) overlaid. In both hemispheres, place and people recall are clustered along a posterior-anterior trajectory, whereas object-recall is far more distributed throughout MPC. (4) The legend for Figure 5 says there should be an anatomical region outlined in white which does not appear to relate to this figure (Potentially Figure 1D?). It is unclear whether the bar graph in this figure relates to the anatomical ROI defined in Figure 1D or the region of functional activation shown in this figure. Additionally, there does not appear to be any overlap on the surface between the small cluster identified in Figure 4A as pSTS and the cluster labelled in Figure 5 as pSTS. Could the authors please clarify exactly how the ROI for this analysis was determined? Response: We apologize for any confusion. The outline of the STS in Figure 1D is simply for reference. For data that comprise Figure 5 were taken from the group-based ROI shown in Figure 5. The reviewer is correct that the small cluster we identify in the pSTS of Figure 4A does not overlap with the independent group ROI but we wanted to avoid any circularity in how we defined this ROI.

Minor Lines 17 and 37: Typo "recruited during multiple cognitive." - Fixed Line 127: Please add upper and lower limits for age. - Done Line 151: Post-scan questionnaire is discussed here but nowhere else in the manuscript. Was this data used in any analysis? Were any trials excluded if the participants could not visualize them? - These data were collected but not used in the analyses. We can confirm that no trials were excluded.

Line 385: What are the units of the scale bar in Figure 4A? - F stat for the main effect of Category. This has been added to figure legend in the revised manuscript.

Line 443: The Object area described in parietal cortex is comparable to a region commonly associated with visual presentation of tools/small manipulable objects (Chao and Martin 2000; Mahon, Schwarzback and Caramazza, 2010). - We have added these citations to the discussion of this aspect of the data.

Line 562: While the specific examples of types of objects given are reasonably recent inventions, the use of tools/hand manipulable objects encompasses >2 million years of human history so this seems an unlikely explanation. - This point is well taken, we now include this alternative explanation in the discussion.

The addition of Bayes Factor analysis may help strengthen the interpretability of the negative findings. - We acknowledge the suggestion. However, we believe that the additional analyses performed, which include showing unthresholded contrast maps of each category versus baseline and a detailed topographical depiction of the spread of peak responses to each category throughout MPC provide strong evidence in favor of the negative findings regarding object-recall effects in MPC.

Is it possible to provide MNI/Talairach coordinates for any of the identified clusters? Will the authors be uploading any of their group maps or datasets to a publicly accessible repository? - These data were analyzed within each participant's native space and then projected to the cortical surface in order for group-level surface data to be computed. In SUMA, these surfaces respect the individual gyral and sulcal patterns of the participants. We therefore would prefer not to convert these into a standard volumetric space. Group-level surface maps will be made available via the Open Science Framework (OSF) upon publication.

Reviewer #2 :

Kidder and colleagues report an fMRI study that examines the brain response during recall of stimuli from different categories including people, objects, and places. In a previous study they found category-selective areas during recall in PMC for people and places. Here they asked whether an object area will be also revealed, similar to the functional organization of faces, objects and places in VTC. Results replicated previous findings with people and places but did not reveal an object region in a nearby location. Object recall activated other brain regions that were not predicted a-priori. The authors conclude that the functional organization during recall in PMC does not mirror the VTC during stimulus presentation.

The paper is well written and clearly presents the motivation for the study, the design and the findings. The data primarily replicates their previous findings that were published in a 2019 paper, but do not reveal much beyond that. Absence of findings does not necessarily indicate that such a region does not exist - it may require a different methodology in order to reveal it. Object regions in VTC are different from face and place areas in that they are typically contrasted with scrambled images. This may be harder to reveal in a recall task. Other than that, beyond the significance of the replication itself, I am not convinced that there is much additional information in the current paper beyond their previous publication. Overall, this is a high-quality paper. My main concern is about its novelty.

Response: We thank the reviewer for their positive appraisal of our work. We acknowledge that object responses in VTC are typically identified via comparing objects versus scrambled objects and that a similar recall contrast would be challenging.

Reviewer #3 :

The authors report an fMRI investigation aimed at revealing the organisation of medial parietal regions that may be involved in the recall of familiar information from verbal cues. Specifically, they built closely on previous work of theirs that identified a a regular anatomical distinction between MPC regions that were activated by the recall of places vs recall of familiar people. In the present study, that work was extended by asking about the recall of familiar objects, with the expectation that MPC might show object-sensitive regions intermediate to place and people regions, mirroring the organisation of human ventral temporal cortex. However, while the people and place regions were replicated, the predicted pattern for objects was not revealed in MPC. The authors further explored for regions in the rest of the brain that were associated to recall of specific categories, offering some post-hoc accounts of their potential significance.

The manuscript is well-organised and well-argued, and builds carefully on previous research. There are good examples of rigor here in the use of independent a priori ROIs for some of the analyses, and in the indirect tests of possible eye movement confounds with MVPA of the eyeball region.

Response: We are grateful for the constructive comments.

While rigorous, the present study does not offer much that is new in the way of theoretical implications, which I take to be a key standard for publication in JN. Replicating parts of the previous findings is of course valuable, and helps confirm the reality of the previous findings but does not particularly inform their significance. The findings of wider regions implicated in memory for people, places, and objects are potentially interesting, but as they were not necessarily expected, they can only be evaluated in an ad-hoc way in the absence of further tests to distinguish amongst possible explanations for how these regions are contributing to behaviour. Finally, as Reviewer 1 hints at and Reviewer 2 notes strongly, null effects are notoriously difficult to interpret; indeed there may be object-related representations in MPC that are simply at a different spatial scale than the others, or that require a different baseline condition to be revealed.

Response: We take the reviewers point here. As R1 requested, we have no added surface-level figures showing the distribution and peaks of people-, place- and object-recall relative to baseline at the group-level, as well as, the location of the peaks for each type of recall in individual participants.
